# A classification scheme of active faults in engineering

**DOI:** 10.1371/journal.pone.0318504

**Published:** 2025-02-07

**Authors:** Qingyun Zhou, Suge He, Zhenyu Zou

**Affiliations:** 1 Yunnan Earthquake Agency, Kunming, Yunnan, China; 2 Kunming Institute of Earthquake Forecast, China Earthquake Administration, Kunming, Yunnan, China; 3 Institute of Earthquake Forecasting, China Earthquake Administration, Beijing, China; Ud'A: Universita degli Studi Gabriele d'Annunzio Chieti Pescara, ITALY

## Abstract

Fault displacement hazard, along with ground shaking hazard and earthquake-induced geohazard, are the primary forms of disaster in major earthquakes. Buildings located on areas of strong seismic surface displacement are likely to be damaged if anti-displacement design is not carried out. Therefore, a reasonable and targeted active fault classification scheme is helpful for avoidance and anti-displacement hazard of active fault in engineering construction. However, the existing classification schemes are rough, and some have no quantitative classification basis, which makes it difficult to apply these classification schemes in actual work. Also, they did not specify whether all active faults should be avoided. In this paper, considering the physical mechanism of earthquakes, using two activity parameters of active faults, “strong earthquake recurrence period” (*T*_RP_) and “strong earthquake elapsed time ratio” (*R*_et_), and referring to the probabilistic seismic hazard analysis method (PSHA), the maximum magnitude of potential earthquake on the fault under different exceedance probabilities (EP) is calculated, and was divided into six levels. The fault displacement hazard level under different exceedance probabilities may be different. For buildings with different importance levels, we recommend six hazard classification schemes with different exceedance probabilities. Standard buildings should avoid active faults with a fault displacement hazard level of Ⅰ ~ Ⅲ (faults that can generate earthquakes of magnitude *m*_0_ and above under a 4% exceedance probability over 100 years). Special buildings and key buildings should avoid active faults with a fault displacement hazard level of Ⅰ ~ Ⅳ (faults that can generate earthquakes of magnitude *m*_0_-0.5and above under a 1% exceedance probability over 100 years). The fault displacement hazard classification scheme given in this paper takes into account the physical mechanism of earthquake occurrence and the importance of buildings, which makes this classification scheme both scientific and practical, helps provide technical support for the design and construction of buildings. This study is still quite preliminary, and there are many issues that need further study.

## 1. Introduction

The destructive effects of major earthquakes on buildings are mainly manifested in three aspects: ground shaking hazard, earthquake-induced geohazard, and fault displacement hazard [1–6]. For the first two types of destructive effects, we can achieve the goal of reducing hazard through some engineering means, and the assessment and treatment methods for these two types of destructive effects are relatively mature. However, for fault displacement hazard, existing research is not yet in-depth. Therefore, in the engineering field, the prevention of fault displacement hazard has always been of great importance. In most seismic design codes, buildings must be away from active faults at a certain distance [7–11]. Those buildings planned to be within the range of the active fault avoidance zone will also be excluded.

The term “active fault” was first proposed by American geologist Lawson [12] when he studied the 1906 San Francisco earthquake with a magnitude of 8.3. Later, different scholars and institutions defined the concept of “active fault” [13–23]. Several studies have demonstrated that active faults are often categorized by their temporal activity, including Neotectonic, Early Pleistocene, Middle Pleistocene, Late Pleistocene, and Holocene faults, as outlined in global research. For instance, Galadini et al. [21]and Wallace [24] discuss active faults in a temporal framework that spans from the Late Pleistocene to the Holocene. These definitions are somewhat consistent, i.e., an active fault is one that has experienced inherited movement and displacement within the recent geological time (e.g., Quaternary, late Pleistocene, Holocene), and is likely to regenerate or continue to move and displace in the near future [1–3,23,25–28]. Many countries have active fault distributions, such as the San Andreas Fault in the United States, the Median Tectonic Line active fault system in Japan, and the Longmenshan Fault in China, etc. [29–31].

Different active faults have different activity levels. The future activity level of a fault can be described at multiple directions. For example, based on the time of the last earthquake that displaced the surface, the active faults were divided into neotectonic faults, early Pleistocene faults, middle Pleistocene faults, late Pleistocene faults, and Holocene faults [26,32–38]. Worldwide, several works have mapped faults and developed databases that integrate and organize structural-geological information from published and unpublished sources to support seismotectonic studies. For example, the QUIN 1.0 and 2.0 databases [39,40], as well as the contributions by Maldonado et al. [41] and Williams et al. [42], provide critical resources for understanding deformation and seismic hazard. However, compared with the 100-year design life of buildings, the division in this time scale is relatively rough. Therefore, some scholars divide the active faults into several levels based on the slip rate [36,43–46]. Also, the active faults can be divided into several levels based on the recurrence period of strong earthquakes of active faults [47,48]. These classifications distinguish active faults with different levels to a certain extent. However, on the one hand, these classifications have no clear basis or physical meaning, and on the other hand, their usage are not given. Therefore, these classifications cannot provide guidance and technical support for engineering construction. In the current building design work, all seismic design codes only require buildings to avoid active faults but fail to distinguish how buildings with different importance should avoid active faults with different activity levels.

Buildings have a corresponding service life. According to the “Uniform standard for design of civil building” (GB 50352-2019) in mainland China, the design service life of ordinary buildings is 50 years, and the design service life of particularly important buildings is 100 years. From the perspective of qualitative analysis, compared with the 11.7 ka of the Holocene faults and the 129 ka of the late Pleistocene faults [49], most active faults may not have experienced high seismic hazard during the whole service life of the building. For buildings, if all active faults (generally referred to late Pleistocene faults and Holocene faults in this study) are considered to have a certain fault displacement hazard and the hazard need to be considered, this will increase the construction cost and reduce the land area that we can use, thus affects the development of the city. Therefore, it is necessary to reclassify the fault displacement hazard based on the importance of buildings and the activity level of active faults.

In this study, considering the physical mechanism of earthquakes, using two activity parameters of active faults, “strong earthquake recurrence period” (*T*_RP_) and “strong earthquake elapsed time ratio” (*R*_et_), and referring to the probabilistic seismic hazard analysis method [50–56], we calculated the fault displacement hazard in several common exceedance probabilities, and divide the fault displacement hazard levels.

## 2. Methods

Paleoseismic methods are used to obtain the recurrence period and elapsed time ratio of strong earthquakes on active faults. Based on these two fault activity parameters, the annual rate of strong earthquakes in the next 100 years was calculated. The maximum earthquake magnitude of the active fault under different exceedance probabilities was calculated referring to PSHA method, and the displacement hazard levels of the active faults were divided.

### 2.1. Recurrence period and elapsed time ratio

The history of human civilizations is very short, which prevents us from determining the recurrence period of strong earthquakes on active faults through historical documents. Among the current methods for determining strong earthquake recurrence periods, the paleoseismic is the main and most reliable method [28]. In paleoseismic research on active faults, while obtaining the recurrence period data (*T*_RP_) of strong earthquakes on faults, we can also obtain the elapsed time ratio (*R*_et_) of strong earthquakes.

Near the intersection of the building and the active fault, a suitable location was selected, and paleoseismic trenches were excavated. AMS-^14^C or other chronological testing methods were used to obtain the sedimentary age of the strata related to the paleoseismic events and thus the time of each paleoearthquake (*t*_1_, *t*_2_,... *t*_n_). The recurrence period of strong earthquakes on active faults is


$$T_{\text{RP}}=\frac{t_{1}-t_{n}}{n-1}$$ (1)

This equation can only represent an estimate of the return period assuming. Such an estimate is affected by uncertainty which is as larger as less are the amount of data. The elapsed time ratio of strong earthquake (*R*_et_) is the ratio of the elapsed time since the last strong earthquake to the recurrence period of strong earthquakes. According to the degree of importance, The design life of residential buildings in China and other countries generally does not more than 100 years [57,58]. Therefore, we set the elapsed time ratio of strong earthquake as


$$R_{\text{et}}=\left(t_{n}+100\right)/T_{\text{RP}}$$ (2)

The parameter *R*_et_ is actually a non-dimensional number. When it is less than 1, especially when it approaches 0, it means that the fault is still accumulating energy, and the fault displacement hazard is relatively low at this time. When it exceeds 1, it means that the fault has accumulated enough energy, so the fault displacement hazard level is relatively high.

### 2.2. Annual rate of strong earthquakes

Based on the recurrence period of strong earthquakes, we can obtain the mean annual rate of strong earthquakes on the fault


$${\bar{\nu }}_{0}=\frac{1}{T_{\text{RP}}}$$ (3)

The term “strong earthquakes” here does not specifically refer to earthquakes greater than a certain magnitude, but rather to those that can cause surface displacement on active faults. Therefore, we recommend using trenching or drilling methods to obtain *T*_RP_ data for active faults. The magnitude of a “strong earthquake” may vary for different active faults in different regions. For a given active fault, the occurrence of strong earthquakes is not evenly distributed over time. After a strong earthquake, the energy accumulated in the Earth’s crust is released, and the probability of strong earthquakes decreases; over time, the energy in the Earth’s crust gradually increases, and the incidence of strong earthquakes increases. Considering the linear increase in the energy of the crust over a long time, combined with the annual rate of strong earthquakes obtained by the paleoseismic, we use the following equation to describe the incidence of strong earthquakes:


$$\nu_{0}\text{=}\left\{\begin{array}{l} 2{\bar{\nu }}_{0}\times R_{\text{et}}\;0<R_{\text{et}}\leq 1\\ 2{\bar{\nu }}_{0}\;R_{\text{et}}\geq 1 \end{array}\right.$$ (4)

According to the above formula, during a strong earthquake recurrence period, the average occurrence rate of $\nu_{0}$ is ${\bar{\nu }}_{0}.$ When the elapsed time ratio of strong earthquake is bigger, this equation can reflect the increased incidence of strong earthquake to some extent.

### 2.3. Exceeding probability of earthquakes with different magnitudes

Compared to *T*_RP_ that are hundreds or thousands of years, the design lifespan of buildings is no more than 100 years [57–59]. Within the 100 years, it can be considered that the incidence of strong earthquakes on faults is constant and unchanging. Therefore, we chose the Poisson process instead of the non-stationary Poisson process. Using the probabilistic seismic hazard analysis method, the probability of *n* earthquakes occurring on the fault in the next year *t* is calculated as follows:


$$P(n)=\frac{{\left(\nu_{0}t\right)}^{n}}{n!}e^{-v_{0}t}$$ (5)

The seismicity on the active faults follows the magnitude–frequency relationship:


$$\log_{10}N(m)=a-bM$$ (6)

*b* in the formula, that is, the *b*-value, reflects the relationship between the quantity of earthquakes with different magnitudes within a certain space [60]. The *b*-values are different in different regions, and even in the same region, the *b*-values calculated by different researchers are also different. In the subsequent calculations, we use the *b*-value of a certain region instead of the *b-*value on the fault.

According to the above two equations, the probability density function of earthquake with different magnitudes can be obtained as


$$f(m)=\frac{\beta \exp[-\beta (m-m_{0})]}{1-\exp[-\beta (m_{\text{uz}}-m_{0})]}$$ (7)

where β=*b*ln10, the upper limit of the magnitude of the fault is *m*_uz_, and the minimum magnitude of an earthquake revealed by a paleoseismic trench (that is, the minimum magnitude that can generate surface displacement) is *m*_0_. The probability of an earthquake with a magnitude less than *m*_0_ can be derived from the probability of an earthquake with a magnitude above *m*_0_. In actual work, magnitude *m* is divided into *N*m bins, and *m*_*j*_ represents the magnitude bin in the magnitude range (*m*_*j*_ ± ∆*m*/2). Then, the annual incidence of earthquakes with magnitude *m*_*j*_ within the fault is:


$$P(m_{j})=\frac{2}{\beta }f(m_{j})\cdot \sinh(\frac{1}{2}\beta \Delta m)$$ (8)

The number of earthquakes with magnitude *m*_*j*_ within the fault in the next *t* year is


$$n_{j}(t)=P(m_{j})\cdot \nu_{0}\cdot t$$ (9)

The number of earthquakes above magnitude *m*_*j*_ within the fault in the next *t* year is


$$m(m_{j},t)=\overset{m_{\text{uz}}}{\underset{j}{\sum }}n_{j}(t)$$ (10)

The probability of at least one earthquake exceeding magnitude *m*_*j*_ in the next *t* year is


$$P(m_{j},t)=1-\exp[-m(m_{j},t)]$$ (11)

### 2.4. Fault displacement hazard classification

According to the major seismic design codes [57–59], we considered the classifications of active faults under the following exceedance probabilities: 1% over 100 years (with a return period of 9950 years), 2% over 100 years (with a return period of 4950 years), 4% over 100 years (with a return period of approximately 2450 years), 5% over 100 years (with a return period of 1950), 10% over 100 years (with a return period of 950 years), and 19% over 100 years (with a return period of 475 years).

The minimum magnitude of an earthquake capable of causing surface displacement (*m*_0_) is controlled by various factors, such as the thickness of the surface cover layer [61,62], and the characteristics of the seismic wave spectrum [63,64]. The value of *m*_0_ may vary across different regions, and even within the same region, it may differ among different faults. Furthermore, different segments of the same fault may also have different *m*_0_ values. For instance, for mainland China, the *m*_0_ in the western part is approximately 6.5, while in the eastern part, it is around 7.2 (due to the significantly thicker overburden in the east compared to the west) [65]. In this study, we take *m*_0_ = 7.0 for example to investigate the classification of faults. For a specific active fault, it is necessary to use paleoseismic methods in conjunction with geophysics, seismology, and geology to comprehensively determine *m*_0_. If the annual occurrence rate of strong earthquakes is obtained using methods such as trenching or drilling, then the size of the *m*_0_ value will not affect the results of the fault displacement hazard classification. Additionally, we divided the fault displacement hazard levels into 6 grades, with an interval of 0.5 magnitude (Table 1), because research on the empirical relationship between the ground shaking levels (seismic intensity) of earthquakes in the Chinese mainland and their magnitudes indicates that for every 0.5 increase in magnitude, the epicentral ground shaking level increases by approximately 1 degree [66].

**Table 1:** Grading criteria for fault displacement hazard level.

Displacement hazard level	Ⅵ	Ⅴ	Ⅳ	Ⅲ	Ⅱ	Ⅰ
Maximum magnitude of potential earthquake	<6.0	6.0–6.5	6.5–7.0	7.0–7.5	7.5–8.0	>8.0
Ground shaking level near epicenter [66]	<Ⅶ*	Ⅷ	Ⅸ	Ⅹ	XI	>XI
Displacement hazard	None	Low	Low-moderate	Moderate	Moderate-high	High

*The division rules can be viewed here: https://gb18306.net/detail/59

## 3. Results

The value of *m*_0_ used in our calculations is 7. If the actual *m*_0_ of an active fault is less than or greater than this value, the magnitudes in the results below should be correspondingly reduced or increased.

### 3.1. Exceedance probabilities of different magnitudes

**Fig 1:**
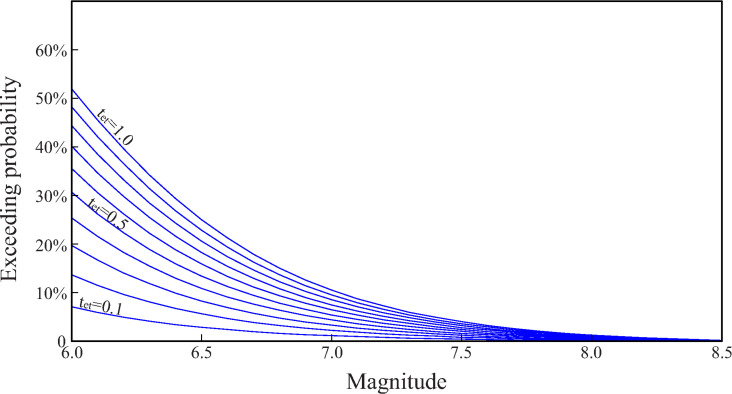
Exceedance probabilities (over 100 years) of earthquakes of different magnitudes on an active fault (*T*_RP_ = 2000 a) under different elapsed time ratios.

We calculated the exceedance probabilities of different earthquake magnitudes on an active fault (*T*_RP_ = 2000a) with different elapsed times ratio (*R*_et_ = 0.1 ~ 1.0) of strong earthquakes (Fig 1). It can be seen that, under different *R*_et_, the exceedance probabilities of an earthquake occurrence are quite different. Taking the exceedance probability of an earthquake with magnitude 6 over 100 years as an example,

if *R*_et_ = 0.1, the exceedance probability is 7%;

if *R*_et_ = 0.5, the exceedance probability is 30%;

if *R*_et_ = 1.0, the exceedance probability is 52%.

This result is also consistent with the physical mechanism of earthquake occurrence: after an earthquake, the accumulated energy on the fault decreases, resulting in a low probability of a major earthquake; as time goes on, the accumulated energy on the fault increases, and the probability of a major earthquake significantly rises. Another example, if a building considerate a 4% exceedance probability over 100 years, then,

if *R*_et_ = 0.1, the maximum earthquake magnitude is 6.3;

if *R*_et_ = 0.5, the maximum earthquake magnitude is 7.2;

if *R*_et_ = 1.0, the maximum earthquake magnitude is 7.5.

This conclusion is consistent with the “seismic gap” method in earthquake hazard prediction: faults that have recently experienced major earthquakes (non-seismic gaps) have a low probability of experiencing another major earthquake in the short term; faults that have not experienced major earthquakes for a long time (seismic gaps) have a relatively higher probability of experiencing major earthquakes [67]. It can be seen from these two examples that the determination of the elapsed time ratio of strong earthquake (*R*_et_) of an active fault is very important for the seismic design of buildings.

### 3.2. Classification of faults under different exceedance probabilities

**Fig 2:**
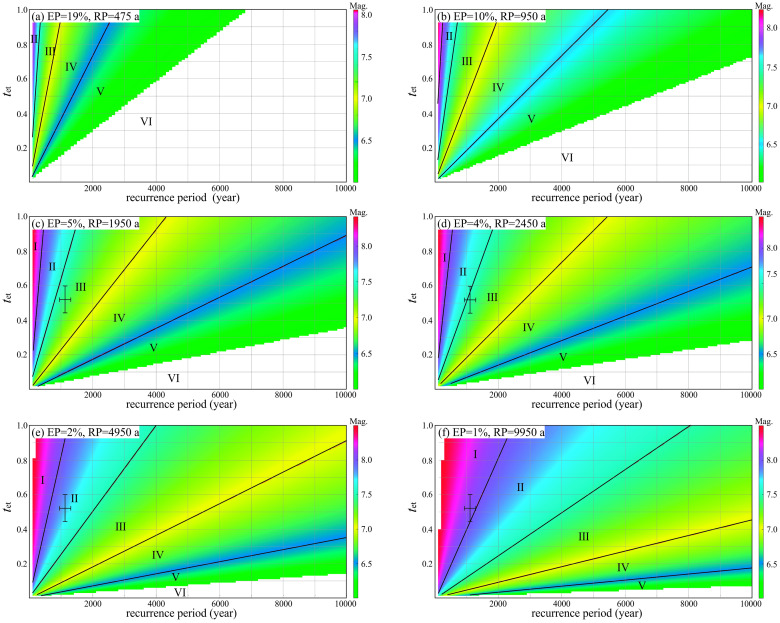
Maximum earthquake magnitude and fault displacement hazard classification with different *T*_RP_ and different *R*_et_ under different exceedance probabilities. EP, exceedance probability over 100 years; RP, return period.

We calculated the maximum earthquake magnitude on active faults with different *T*_RP_ and different *R*_et_ under six commonly used exceedance probabilities (Fig 2). We divided each calculation result into six levels, I-VI, with the hazard levels as high, moderate-high, moderate, low-moderate, low, and none. Based on Fig 2a–2f, the following conclusions are easily drawn:

(1) Under different exceedance probabilities, the fault displacement hazard levels may be different. For example, for active fault with *T*_RP_ = 2000 and *R*_et_ = 0.6: when the exceedance probability is 19% (over 100 years), the hazard level is low; when the exceedance probability is 10%, the hazard level is moderate-low; when the exceedance probability is 4% or 5%, the hazard level is moderate; and when the exceedance probability is 1% or 2%, the hazard level is moderate-high.
(2) Except for very important buildings such as nuclear power plants and very large reservoir dams, most buildings consider only earthquake motion with a return period of 2450 years. The return period of 2450 years (exceedance probability 4% over 100 years) is shown in Fig 2d. In most cases (*T*_RP_ > 5500 years), the earthquake magnitude of an active fault is not expected to exceed 7 with a 4% probability of exceedance over a 100-year period; therefore, the fault displacement hazard is not considered for most buildings in most cases (*T*_RP_ > 5500 years).

Compared with previous studies, our results differ in two aspects. First, we have taken into account the process of energy accumulation on the fault, hence the probability of the fault experiencing a major earthquake is related to *R*_et_. Second, since we have referred to the PSHA method, the expected maximum earthquake magnitude of an active fault varies under different return periods.

## 4. Discussion

### 4.1. Comparison with other classifications

Active faults can be classified according to their slip rate or according to their recurrence period of strong earthquakes. In terms of the slip rate, Slemmons and Depolo [44] divided active faults into six different levels according to their slip rate (Table 1). Chang and Zhang [68], and Wu and Zhou [69] classified active faults according to the fault slip rate. However, these classification scheme have obvious deficiencies. First, these types of classification schemes are aimed at active faults at the global scale and does not consider the difference in the rate of different types of faults inside the continent. Second, these classification schemes are relatively rough. The slip rate of most active faults in continental interior is 1 ~ 10 mm/a, and their hazard levels are all level A (Table 2) [44]. These classification schemes cannot be used to effectively distinguish the fault displacement hazard level in continental interior. Third, it is difficult to accurately differentiate the creep slip rate, stick-slip rate, slow earthquake rate and slip rate on distributed faults. Although we can classify active faults based on their slip rates, the classification results may not be useful for engineering design and construction.

**Table 2:** Classification based on the fault slip rate (modified from Slemmons and Depolo [44]).

Category	Activity	Slip rate (mm/a)	Characteristics of fault activity
AAA	Very high	≥100	The surface manifestations are particularly prominent, with a very high fault activity rate, mainly occurred on the plate boundary, especially the subduction boundary zone
AA	High	100-10	Own prominent surface manifestations and a high fault activity rate, and most occur on plate boundaries or on the active large on type block boundary
A	Moderate-high	10 ~ 1	There are abundant but sometimes discontinuous signs of surface activity and a strong activity rate
B	Moderate	1-0.1	Surface activity markers and moderate activity rate with moderate development or locally better surfaces
C	Low	0.1-0.01	There is less evidence of surface activity and lower activity rate and activity
D	Very low	<0.01	Surface traces of faults are not easy to identify, and activity is very low or basically inactive

The fault recurrence period better reflects the true activity level of the fault. Based on the recurrence period of strong earthquakes on faults, Kerr et al. [47], Litchfield et al. [70], Christophersen et al. [35], Wu and Zhou [69], Wu [71,72], Wu and Hu [73] proposed classification schemes of active fault levels. These classification schemes may better reflect the activity of the faults. However, these classification schemes are mainly for scientific research and do not consider the application in building construction. For example, Litchfield et al. [70] and Wu [72] classified active faults with *T*_RP_ ≤ 100 years as hazard level 1, which less than the design service life of the building. At the same time, these classification schemes did not provide the indicators and reasons for the classification, nor did they consider the physical mechanism of earthquakes. Some researchers have considered *T*_RP_ and *R*_et_ in fault hazard classifications, but they simply use the ratio *R*_et_/*T*_RP_ as the classification standard to classify the hazard of faults [72,74]. Fig 2 shows that the ratio classification scheme ignores the differences among faults with different *T*_RP_, which underestimates the hazard of active faults with shorter *T*_RP_ and overestimates the hazard of active faults with longer *T*_RP_.

**Fig 3:**
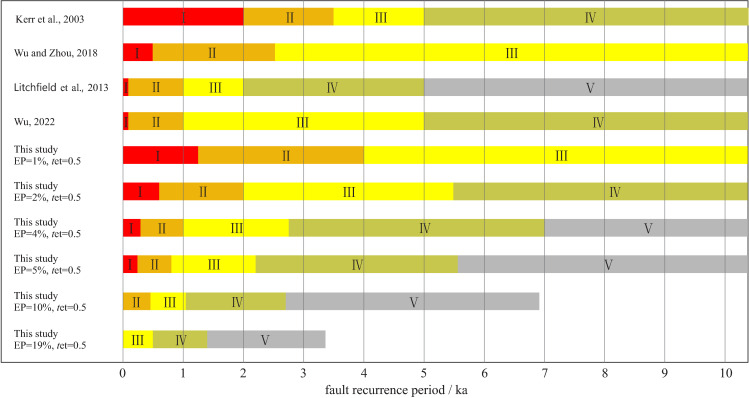
Other researchers’ classification results and the classification results of this paper under different exceedance probabilities.

Our classification results are shown in Fig 2. We extracted the fault displacement hazard levels when *R*_et_ = 0.5 under different exceedance probabilities from Fig 2 and compared these results with other researcher’s hazard level classification results based on the recurrence period of strong earthquakes (Fig 3). The classification result given by Kerr et al. [47] is close to the result of EP = 1% in this paper. The classification result given by Wu and Zhou [69] is close to the result of EP = 2% in this paper. The classification result given by Litchfield et al. [70] is close to the result of EP = 4 ~ 5% in this paper. However, this “close to” is only local similarity, not similarity in the overall trend. The reason is that in the past, the hazard classifications of active faults were mostly based on researchers’ own experience, and the classification results did not have clear physical meaning.

### 4.2. Late Pleistocene faults

If a fault experienced earthquakes that displaced the surface in the Late Pleistocene but not experienced such earthquakes in the Holocene, this type of fault is called late Pleistocene fault. While it is unlikely that Late Pleistocene faults generate earthquakes with magnitude 7 or above, it cannot be excluded that these faults may reactivate under certain tectonic conditions. Compared with the Holocene fault, the *T*_RP_ of the Late Pleistocene fault is much longer, and thus, the fault displacement hazard is much lower. In the seismic design codes, the possibility of surface displaced earthquakes in the Late Pleistocene is not considered in most cases except for particularly important buildings and structures such as nuclear power plants and very large dams [75,76].

**Fig 4:**
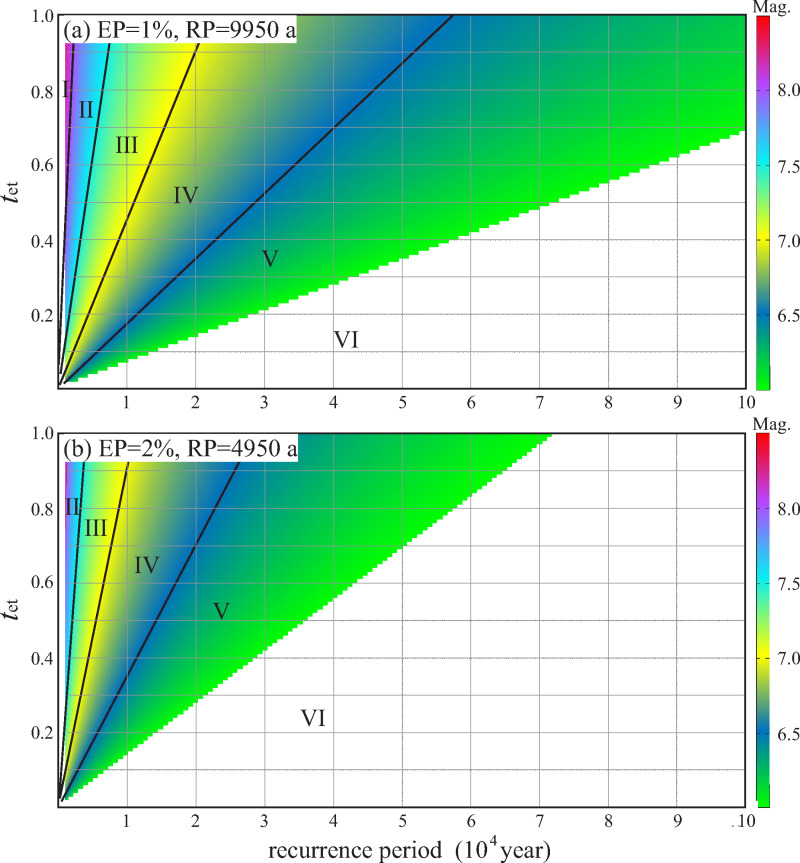
Maximum earthquake magnitude and displacement hazard classification of active faults with different strong earthquake recurrence periods and different strong earthquake elapsed time ratios on the conditions of exceedance probability 1% and 2% over 100 years.

If given enough time, it is inevitable that earthquakes of magnitude 7 or greater will occur on late Pleistocene faults, as long as the scale of the fault is large enough. However, if time is limited, it is not certain that Late Pleistocene faults can produce earthquakes of magnitude 7 or greater. Fig 2d shows that, when considering the seismic hazard of return period of 2450 years, the fault displacement hazard level of faults with *T*_RP_ ≥ 5500 years will never exceed Level IV; that is, an earthquake with a magnitude of 7 or above will not occur on the faults. Therefore, the statement that “the Late Pleistocene faults do not have the tectonic conditions for earthquakes with magnitude 7 and above” is correct and conservative according to this study. The design life of ordinary buildings and structures is no more than 100 years [57–59], and the maximum return period that should be considered in seismic design of buildings is 2450years; therefore, the influence of Late Pleistocene faults does not need to be considered. However, for particularly important buildings and structures such as nuclear power plants and very large dams, the fault displacement hazard with a return period of 4950 years (exceedance probability 2% over 100 years) or 9950 years (exceedance probability 1% over 100 years) also needs to be considered. Therefore, we calculated the maximum potential earthquake magnitude of Late Pleistocene faults with different *T*_RP_ and *R*_et_ under the two exceedance probabilities and classify the fault displacement hazard levels accordingly (Fig 4).

When the exceedance probability is 1% over 100 years, late Pleistocene faults with *T*_RP_ more than 22000 years do not have tectonic conditions for the occurrence of earthquakes with a magnitude of 7 and above; when exceedance probability is 2% over 100 years, Late Pleistocene faults with *T*_RP_ more than 11000 years do not have tectonic conditions for the occurrence of earthquakes with a magnitude of 7 and above. For those particularly important buildings, the possibility of surface displacement caused by an M6.5 (*m*_0_-0.5) earthquake is often considered; therefore, the displacement hazard level of the fault should be raised by one level when aim building is very important. For example, an active fault next to a nuclear power plant, the surface displacement hazard level of the fault calculated by the method in this paper is level Ⅳ. Considering the importance of the nuclear power plant, the surface displacement hazard level of the fault should be raised by one level, and be evaluated as level Ⅲ. In this sense, the displacement hazard of Late Pleistocene faults with *T*_RP_ short than 57000 years (exceedance probability 1% over 100 years) and 28000 years (exceedance probability 2% over 100 years) should be attention. Even if the fault displacement hazard level is raised by one level, we do not need to consider the fault displacement hazard with *T*_RP_ longer than 57000 years. Therefore, in the engineering field, the displacement hazard of middle and early Pleistocene faults does not need to be considered.

### 4.3. Displacement hazard of buildings with different importance

According to the different importance levels of the buildings, we divided the buildings into four categories: special buildings, key buildings, standard buildings, and less important buildings. Special buildings refer to buildings that may have particularly large disaster consequences during an earthquake, such as severe secondary disasters during an earthquake, such as nuclear power plants and very large reservoir dams. Key buildings refer to important lifeline projects buildings that may cause a large number of death and other major disaster consequences during an earthquake, such as important bridges, stadiums, schools, and hospitals. Less important buildings are buildings that are rarely used and where an earthquake will not cause secondary disasters, such as ordinary warehouses. Except for the above 3 types of buildings, the other buildings are standard buildings. During the design stage of a building, structures of varying importance typically need to consider seismic actions for different exceedance probabilities. Also, we can judge the importance of a building based on the exceedance probability of the seismic actions it needs to consider (Table 3).

**Table 3:** The relationship between the importance of a building and the exceedance probabilities of seismic actions considered during the design stage.

Building importance	Special buildings	Key buildings	Standard buildings	Less important buildings
Exceedance probability (over 100 years)	1%	2%	4%	19%
Return period (years)	9950	4950	2450	475

For less important buildings, we suggest using the classification result of exceedance probability 19% over 100 years (exceedance probability 10% over 50 years) (Fig 2a). In this scenario, only the strongly active fault that its *T*_RP_ shorter than 1000 years can reach level III. Except for very few faults located in special tectonic locations, the recurrence period of strong earthquakes on most active faults inside the plates is longer than 1 ka. Therefore, in most cases, less important buildings do not need to avoid active faults. This is consistent with various current seismic design codes.

For standard buildings, we suggest using the classification result of exceedance probability 4% over 100 years (exceedance probability 2% over 50 years) (Fig 2d). In this scenario, the fault displacement hazard level with a *T*_RP_ longer than 5700 years are lower than level III, and the fault displacement hazard level with a *T*_RP_ shorter than 2000 years may reach level II. Considering the *T*_RP_ of continental interior faults are longer than 100 years, it is less likely that the fault displacement hazard level can reach level I.

For key buildings, because this type of building may cause a large number of death during an earthquake, we recommend using the classification result of exceedance probability 1% over 100 years (Fig 2f, Fig 4a). In this scenario, when the fault’s *T*_RP_ is shorter than 22000 years, the fault displacement hazard level may reach level III; when the fault’s *T*_RP_ is shorter than 8100 years, the fault displacement hazard level may reach level II; when the fault’s *T*_RP_ is shorter than 2400 years, the fault displacement hazard level may reach level Ⅰ.

For special buildings, if this type of building is damaged in an earthquake, it may cause extremely large number of death. Therefore, we recommend using the classification result with exceedance probability 1% over 100 years (Fig 2f, Fig 4a), and based on this, the fault displacement hazard level should be raised by one level. The largest *T*_RP_ for active faults that can reach levels III, II and I are 57000 years, 22000 years and 8100 years, respectively.

### 4.4. Significance of different fault displacement hazard levels

In the previous classification schemes, the fault displacement hazard level has no actual physical meaning. This paper uses the probabilistic seismic hazard analysis method and the earthquake magnitude as the grading standards to divide the fault displacement hazard into levels I ~ VI from high to low, and each level has an actual physical meaning (Table 4).

**Table 4:** Physical meaning, avoidance suggestions and fault naming of each fault displacement hazard level.

Hazard level	Potential earthquake magnitude*	Surface displacement	Potential displacement (m)**	Avoidance suggestion	Fault naming
I	≥8/ ≥ (*m*_0_ + 1)	Enormous surface displacement	≥12.6	A-D*** should strictly avoid	Great Hazard Fault
II	7.5–7.9/(*m*_0_ + 0.5 to *m*_0_ + 0.9)	Moderate surface displacement	4.9–10.4	A-C should avoid.	Hazard Fault
III	7.0–7.4/(*m*_0_ to *m*_0_ + 0.4)	Can generate surface displacement	1.9–4.1	A-B should avoid.	
IV	6.5–6.9 (*m*_0_-0.5 to *m*_0_-0.1)	Surface displacement may occur	0.7–1.6	A should avoid.	Perhaps hazard Fault
V	6.0–6.4 (*m*_0_-1.0 to *m*_0_-0.6)	No surface displacement	/	No need to avoid	No hazard Fault
VI	<6.0 < (*m*_0_-1.1)	No surface displacement	/	No need to avoid	

*If the minimum magnitude of earthquakes that can cause surface displacement on the target active fault is not 7.0, then the potential earthquake magnitude should also be correspondingly increased or decreased.

**The expected displacement calculated based on the empirical relationship between magnitude and surface displacement given by Wells and Coppersmith [77].

***A refers to special buildings; B refers to key buildings; C refers to standard buildings; D refers to less important buildings.

Level I: In the next 100 years, there is a certain probability that at least one earthquake with a magnitude of 8.0/(*m*_0_ + 1) and above will occur. Active faults at this level are uncommon. Only when the recurrence period of strong earthquakes is less than a certain value and the return period of ground motion considered in buildings is very large can the fault displacement hazard level reach level I. When the fault displacement hazard level reaches level I, we suggest that this type of fault should be strictly avoided.
Level II: In the next 100 years, there is a certain probability that at least one earthquake with a magnitude of 7.5 ~ 7.9/(*m*_0_ + 0.5 to *m*_0_ + 0.9) will occur. When the fault displacement hazard reaches level II, all buildings except for less important buildings should avoid level II active faults.
Level III: In the next 100 years, there is a certain probability that at least one earthquake with a magnitude of 7.0 ~ 7.4/(*m*_0_ to *m*_0_ + 0.4) will occur. These earthquakes can generate permanent surface displacement. Therefore, except for less important buildings, buildings should avoid level III active faults.
Level IV: In the next 100 years, there is a certain probability that at least one earthquake with a magnitude of 6.5 ~ 6.9/(*m*_0_-0.5 to *m*_0_-0.1) will occur. An earthquake of this magnitude may or may not generate surface displacement, depending on factors such as the focal depth, earthquake type, and overburden thickness. Therefore, we recommend that special buildings should avoid level IV active faults.
Level V: In the next 100 years, there is a certain probability that at least one earthquake with a magnitude of 6.0 ~ 6.4/(*m*_0_-1.0 to *m*_0_-0.6) will occur. An earthquake of this level will not cause surface displacement; therefore, all buildings do not need to avoid level V active faults. However, the near-fault effect and the hanging wall effect need to be considered.
Level VI: In the next 100 years, there is a certain probability that at least one earthquake with a magnitude of 5.9/ (*m*_0_-1.1) and bellow will occur. For an earthquake of this magnitude, the intensity near the epicenter is generally 7–8 degrees (PGA = 0.10–0.20g), which does not exceed the seismic fortification intensity of buildings. Therefore, level VI active faults are not dangerous.

### 4.5. Distributed rupture and kinematics

**Fig 5:**
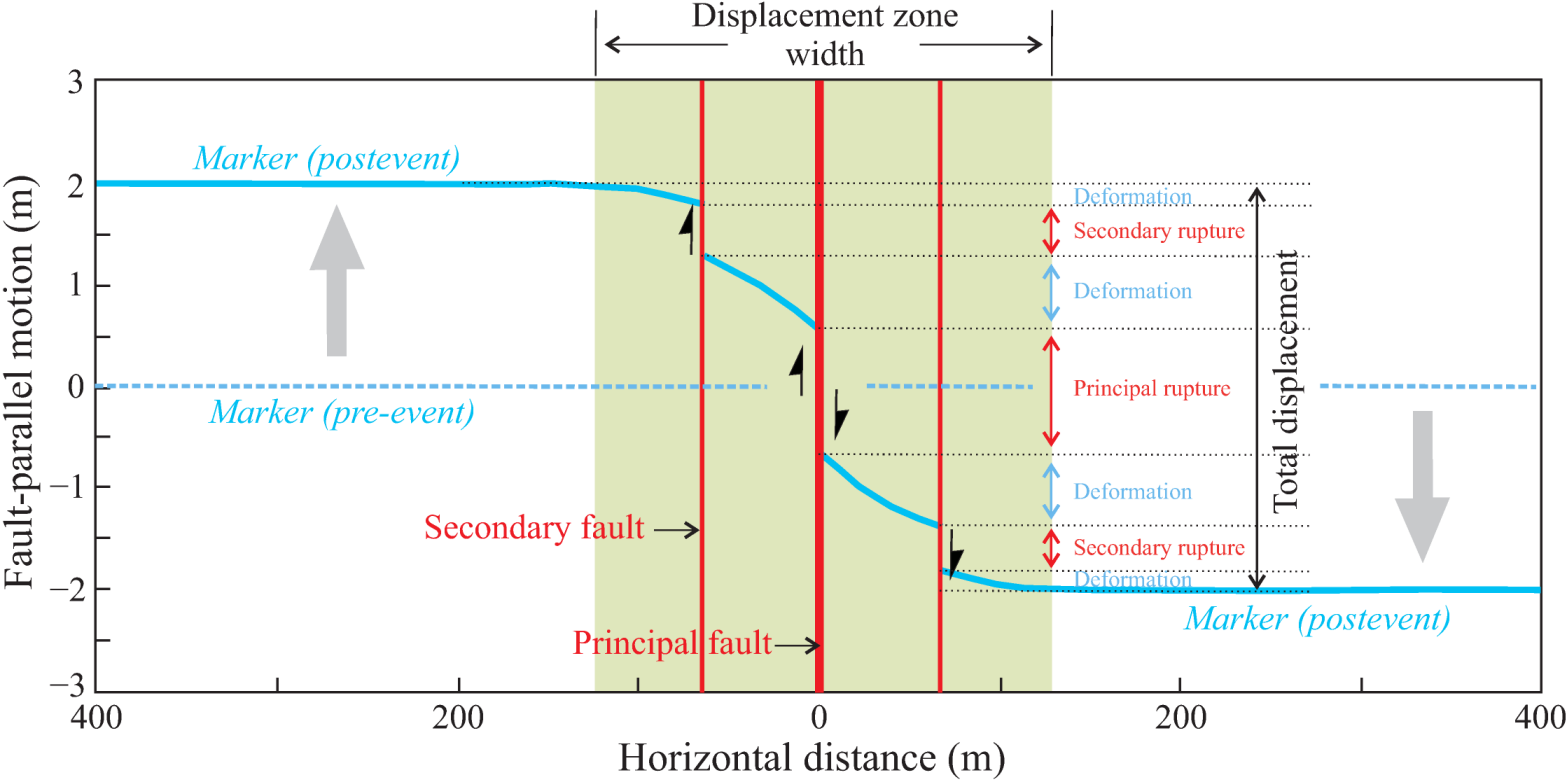
A sketch showing the relationship between the rupture that is localized on the fault plane, the deformation between faults, and the total surface displacement. PD, principal rupture; DD, secondary rupture + deformation.

In a major earthquake, co-seismic surface displacement is not only distributed along the principal fault (principal displacement, PD), but may also exist within a certain distance from the principal fault (distributed displacement, DD). Many scholars have observed this phenomenon and conducted various studies (e.g., [78–84]). From the perspective of earthquake engineering, we categorize co-seismic surface displacement into two types: ruptures and deformations. Ruptures refer to surface displacement caused by the relative rupture of the hanging wall and footwall of a fault (including the principal fault and secondary faults). Deformation refers to surface displacement between faults (Fig 5). Existing research has shown that in major earthquakes, the proportion of DD is approximately (50 ± 15)% of the total displacement [85,86]. Although the proportions of DD and PD are roughly equivalent, ruptures occur within a relatively narrow spatial range (a few centimeters to a few meters), while deformations occur within a relatively wide spatial range (100 meters to several hundred meters). Therefore, the destructive power of ruptures to buildings is much greater than that of deformations (e.g., [23]). The surface displacement revealed by paleoseismic methods is what we refer to as ruptures. Hence, our fault displacement hazard classification method can accurately reflect the surface displacement hazard of faults to buildings. It should be noted that multiple faults (principal faults and secondary faults) may be exposed in a single trench. We recommend conducting fault displacement hazard classification for each fault.

Unlike the PSHA method, the fault displacement hazard assessment method proposed in this paper is not sensitive to the kinematic properties of active faults. Therefore, both strike-slip faults and dip-slip faults can use this method to assess their fault displacement hazards. It should be noted that when we use the trenching method to obtain the *T*_RP_ of a fault, the width of the trench should be determined according to the kinematic properties of the target fault: the surface displacement width of a strike-slip fault is generally small, and both the hanging wall and footwall are prone to surface displacement. Thus, the trench for detecting a strike-slip fault should extend to both sides of the principal fault. The hanging wall of a dip-slip fault may be more likely to produce surface displacement, so the trench for detecting a dip-slip fault should extend further towards the hanging wall of the principal fault [23]. If conditions permit, in actual work, we recommend that the trench span the entire displacement zone.

### 4.6. Limitations and future works

The method proposed in this paper may not be applicable to all faults. In this study, we used a linear non-stationary Poisson process to characterize the rule of how earthquake occurrence probabilities change over time. Although this is an improvement on the Poisson process after considering the physical process of energy accumulation in the crustal medium, due to the complexity of earthquake mechanisms, the linear non-stationary Poisson process may not accurately depict the mechanisms of earthquakes. For example, the rapid healing of faults observed after large earthquakes using seismological and other methods, and the deviation of the magnitude-frequency relationship from statistical relationships at high magnitudes, all indicate that the linear non-stationary Poisson process in this paper has a certain scope of application. Based on the previous analysis, the method proposed in this paper is not suitable for three types of faults: those that have just experienced a large earthquake, those that clearly do not have a strong earthquake recurrence cycle, and those with a long interval since the last strong earthquake. Here, we cannot define the exact numbers for “just experienced a large earthquake” and “long interval since the last strong earthquake.” In future, researchers may focus on finding a model and method that can more accurately describe the mechanisms of large earthquake occurrence.

High-precision *T*_RP_ data is difficult to obtain. Obviously, if we could obtain high-precision *T*_RP_ data, we would be able to accurately assess the fault displacement hazard. However, due to various factors, such as the uncertainty of dating data, the uncertainty of stratigraphic age, and the uncertainty caused by limited number of data in statistical analysis, we may not be able to obtain high-precision *T*_RP_ data. Taking the latest data of the Red River Fault [87] as an example, we simply calculated the impact of *T*_RP_ uncertainty: the recurrence period of strong earthquakes for the Red River Fault is 960–1320 years, with a mean time to next earthquake (*R*_et_) of 0.44–0.60. The calculation results are shown in Fig 2c–2f. It can be seen that the uncertainty of data still has a significant impact on our evaluation results. Addressing the issue of data uncertainty, future work may focus on two aspects: one is to improve dating methods and statistical analysis to reduce the uncertainty of dating data; and the second is to study the relationship between other activity parameters of active faults and T_RP_, for example, studying how to convert the slip rate of active faults into *T*_RP_.

Strong earthquakes on active faults do not always recur strictly periodically. This is manifested in two aspects. First, strong earthquakes may occur when *R*_et_ = 1, also they may occur when *R*_et_ is between 0.9 and 1.1 (e.g., [88,89]). Second, the recurrence patterns of strong earthquakes on some active faults are not periodic but rather exhibit clustering and anti-clustering (e.g., [90,91]). For the first situation, we can conduct more in-depth research in the future to provide a more reliable probability function for the occurrence of strong earthquakes on active faults. For the second situation, it is necessary to accurately delineate the cluster time and anti-cluster time of the faults, calculate the *T*_RP_ separately, and assess the fault displacement hazard.

## 5. Conclusions

Based on the two activity parameters of the active fault, the recurrence period of the strong earthquake and the elapsed time ratio of the strong earthquake, and by referring to the classic probabilistic seismic hazard analysis method, the maximum potential earthquake magnitude of the active fault under different exceedance probabilities is calculated, and based on that, the fault displacement hazard levels are divided. After analysis, the following conclusions were obtained.

(1) The bigger the elapsed time ratio of strong earthquake is, the greater fault displacement hazard. Therefore, it is very important to obtain the elapsed time ratio of strong earthquake of active faults for the seismic design of engineering buildings.
(2) The active fault displacement hazard classification scheme proposed in this paper takes the physical process of earthquake occurrence into consideration and provides clear classification standards and reasons, so it is more suitable for application in engineering design and construction. This method may not be directly applicable to the assessment of surface displacement hazards for active faults that do not have a strong earthquake recurrence cycle.
(3) The fault displacement hazard with different recurrence periods and different elapsed time ratios of strong earthquakes were calculated under six exceedance probabilities. The fault displacement hazard level may be different under different exceedance probabilities. When determining the fault displacement hazard level, the exceedance probability should be indicated.
(4) For special buildings, key buildings, standard buildings, and less important buildings, we recommend classifying the fault displacement hazard levels under the conditions of exceedance probability: 1% (the hazard level should be raised by 1 level), 1%, 4% and 19% over 100 years. Classification assessment is a prerequisite for the application of fault displacement hazard assessments in actual work.
(5) For active faults with a *T*_RP_ longer than 5500 years, under a 4% exceedance probability over the next 100 years, the fault displacement hazard level will not reach Level III (maximum potential magnitude < *m*_0_). Therefore, we can define “engineering active faults” as “faults that have experienced surface displacement earthquakes since 5500 years ago.” For particularly important structures, such as nuclear power plants, the fault surface displacement hazard under a 1% exceedance probability over the next 100 years must be considered, and it is required that the fault displacement hazard level does not reach Level Ⅲ (after be raised by one level, maximum potential magnitude < *m*_0_ − 0.5). In this case, we can define “nuclear active faults” as “faults that have experienced surface displacement earthquakes since 57,000 years ago.” The time limit for “engineering active faults” is set at 5.5 ka, which is significantly lower than the 40 ka in Italy’s “Seismic Microzonation” and also lower than the 11 ka of American’s A-P act.
(6) Standard buildings should avoid active faults with a fault displacement hazard level of Ⅰ ~ Ⅲ. Special buildings and key buildings should avoid active faults with a fault displacement hazard level of Ⅰ ~ Ⅳ.

Our research in this study is still quite preliminary, and there are many issues that need further study, such as how to solve the problem of high uncertainty in the *T*_RP_ of active faults, how to convert the slip rate of active faults into *T*_RP_, how to obtain accurate *b-*values of active faults, how to obtain more accurate non-stationary Poisson processes to describe the mechanism of earthquake occurrence, how to obtain accurate minimum earthquake magnitude that causes surface displacement, and so on. The fault displacement hazard classification of engineering active faults still requires more researchers to conduct research together.
